# Self-absorption correction on 2D X-ray fluorescence maps

**DOI:** 10.1038/s41598-023-33383-w

**Published:** 2023-05-04

**Authors:** Mingyuan Ge, Hanfei Yan, Xiaojing Huang, Yong S. Chu

**Affiliations:** National Synchrotron Light Source II, Brookhaven National Laboratory, Upton, NY USA

**Keywords:** Materials science, Nanoscience and technology

## Abstract

X-ray fluorescence mapping (XRF) is a highly efficient and non-invasive technique for quantifying material composition with micro and nanoscale spatial resolutions. Quantitative XRF analysis, however, confronts challenges from the long-lasting problem called self-absorption. Moreover, correcting two-dimensional XRF mapping datasets is particularly difficult because it is an ill-posed inverse problem. Here we report a semi-empirical method that can effectively correct 2D XRF mapping data. The correction error is generally less than 10% from a comprehensive evaluation of the accuracy in various configurations. The proposed method was applied to quantify the composition distribution around the grain boundaries in an electrochemically corroded stainless steel sample. Highly localized Cr enrichment was found around the crack sites, which was invisible before the absorption correction.

## Introduction

**Figure 1:**
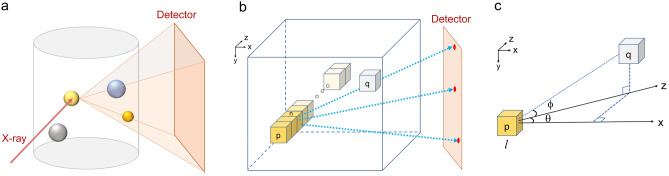
Absorption geometry for scanning XRF mapping. (**a**) A local region of a sample illuminated by a focused X-ray beam produces fluorescence emission in all directions. A portion of the emitted fluorescence photons is collected by a detector. The orange-shaded area indicates the sample volume responsible for attenuating the detected XRF signals. (**b**) Representation of voxels illuminated by the incidence X-ray beam at location p and the detection of the fluorescence photons by the detector through the sample volume. Voxel q represents a local volume of the sample contributing to the absorption of the fluorescence photons. (**c**) the angular description between the emitter of the fluorescence photons along the beam path and an arbitrary voxel within the sample responsible for absorption.

X-ray fluorescence mapping (XRF) is a non-invasive and direct measurement of material composition^1,2^. The recent advances in bright synchrotron sources and X-ray microscopes have significantly enhanced the XRF spatial resolution (~ 10 nm)^3^ and detection sensitivity^4^. Over the past several decades, broad applications of XRF mapping in material research^5,6^, environmental science^7^, and biology^8,9^ have brought massive demands in the quantitative analysis of elemental distribution. In biological or environmental research, quantifying the concentration of the toxic components in cells or plants can provide critical knowledge to understand their biotoxicity and transport pathways^10^. In material science, precise characterization of composition heterogeneity is a prerequisite for understanding the associated properties. Fine-tuning the composition (of Ni, Mn, and Co) and its microstructure (gradient concentration, core–shell, etc.) in Li-ion battery research can significantly improve the structural stability of the cathode material (LiNiMnCoO_2_) and boost the battery performance^11^. However, it is challenging to reliably quantify a spatially resolved composition and establish the link bridging the microstructure and electrochemical behavior. Electron microscopy, despite its atomic resolution and multiple detection capabilities such as fluorescent and electron energy loss spectra^12^, is excellent for small samples but not suitable for high-throughput and statistical analysis on large samples (e.g. > 1 µm). On the other hand, synchrotron-based XRF can image large samples up to hundreds of micrometers, yet the “self-absorption” problem essentially hinders the fully quantitative analysis. The fluorescent emission from the elements can be subject to significant absorption, particularly for materials with high mass density or electron density. As shown in Fig. 1a, the exact amount of absorption is governed by the sample geometry. A complete solution to the self-absorption correction requires knowledge of the geometric details of the sample and the precise amount of attenuation detected.

Over the past several decades, extensive research has focused on recovering the attenuated XRF intensity modified by self-absorption. A few simplifications and hypotheses were proposed to make the correction feasible. For example, correction on the 3D volume can be simplified into corrections on individual 2D slices independently; or assume a uniform attenuation in a weakly attenuated sample^13–16^. However, these methods inevitably compromise the accuracy of the correction since they do not fully incorporate the sample/detector geometry and the heterogeneity of material composition. Recently, we have demonstrated that the self-absorption problem can be solved for 3D XRF mapping without a priori knowledge using an iterative reconstruction engine to achieve self-consistency^6^. However, correcting 2D XRF is still an unsolved challenge since there are insufficient data points to solve this ill-posed inverse problem. A recent study by Ippoliti et al*.* demonstrated using simulated datasets that it is possible to correct attenuation by gathering XRF signals from multiple detectors with a different angular relationship with the sample^17^. This work gains information about the local topology of a homogeneous sample using signal variation from multiple detectors. Here, we propose a correction method using a single detector that corrects the self-absorption on heterogeneous 2D XRF mapping datasets with high fidelity and accuracy. The correction error is estimated to be less than 10% in general. In the following, we formulate the XRF problem and evaluate its accuracy under various cases. We also present an example with experimental data to show how the absorption correction is necessary to reveal the critical material properties that are not visible in the as-acquired 2D XRF mapping data before the absorption correction.

## Method

As schematically represented in Fig. 1, a typical XRF mapping measurement is conducted by raster-scanning a sample in the x–y plane with a focused X-ray beam, illuminating the sample in the z-direction. At a sample position, *p*(*x, y*)*,* XRF emission can occur at any voxels along the z-axis (Fig. 1b), containing multiple constituent elements. However, only a portion of the XRF signal is collected due to the limited acceptance angle of the detector. The detected XRF signal emitted from a specific element *s* by can be evaluated as

1 $${Y}_{det}^{s}\left(p\right)={I}_{0}\sum_{n=0}^{N-1}\left[{A}_{Beam}\left(p,n\right)\cdot {Y}^{s}(p,n)\cdot {A}_{FL}(p,n)\right].$$

${I}_{0}$ is the intensity of the incident X-ray beam. *n* is the voxel index along the z-direction. ${A}_{Beam}$ accounts for the attenuation of the incident beam through the sample. ${Y}^{s}$ is the emitted fluorescence intensity. ${A}_{FL}$ is the attenuation of the XRF signal due to self-absorption. ${A}_{Beam}$ and ${A}_{FL}$ can be written as:

2 $${A}_{Beam}\left(p,n\right)=\mathrm{exp}\left(-nd\sum_{c}\left({r}^{c}\left(p,n\right)\cdot {\mu }_{E}^{c}\left(p,n\right)\right)\right),$$

3 $${A}_{FL}\left(p,n\right)=\underset{\Omega }{\overset{}{\int }}d\sigma \mathrm{exp}\left(-l\left(\sigma \right)\cdot {\sum }_{c}\left({r}^{c}\left(p, \sigma \right)\cdot {\mu }_{FL}^{c}\left(p,\sigma \right)\right)\right).$$

In Eqs. (2) and (3), ${r}^{c}$ is the weight percentage of the constituent element *c* in individual voxels. ${\mu }_{E}^{c}$ is the linear attenuation coefficient for the constituent element *c* at the incident X-ray beam energy. $d$ is the voxel size. ${\mu }_{FL}^{c}$ is the linear attenuation coefficient for element component *c* at XRF energy. Note that ${r}^{c}$, ${\mu }_{E}^{c}$, and ${\mu }_{FL}^{c}$ are functions of spatial coordinate, e.g. at the position of $\left(p, n\right)$ or $\left(p,\sigma \right)$, where $\sigma$ represents the direction of XRF emission within the solid angle $\Omega$ defined by the camera (Fig. 1b). $l\left(\sigma \right)=\frac{d}{\mathrm{cos}\left(\theta \right)\mathrm{cos}\left(\phi \right)}$ is the effective beam path length for the fluorescence photons emitted in direction $\sigma$ see geometric illustration in Fig. 1c.

In the above equations, ${\mu }_{E}^{c}$ and ${\mu }_{FL }^{c}$ can be obtained from an X-ray database (e.g. Xraylib). ${Y}_{det}^{s}\left(p\right)$ and ${I}_{0}$ are obtained from experimental measurements. The task is to find the un-attenuated fluorescence intensity at the location of the fluorescence emission *p*:

4 $${Y}_{emit}^{s}\left(p\right)=\sum_{n=0}^{N-1}{Y}^{s}\left(p,n\right).$$

In the case of 2D projection imaging, the exact information regarding the composition variation along the z-direction is missing. For now, we assume a uniform composition distribution. Note that this assumption can be removed for a 3D volumetric dataset^6^. With the assumption of uniform composition variation through the sample thickness, Eqs. (1), (2), (3) are converted to:

5 $${Y}_{det}^{s}\approx {I}_{0}\cdot \left(\frac{1}{N}\sum_{n=0}^{N-1}{\overline{A} }_{beam}\left(p\right)\right)\cdot {Y}_{emit}^{s}\left(p\right)\cdot {\overline{A} }_{FL}\left(p\right),$$

with

6 $$\sum_{n=0}^{N-1}{\overline{A} }_{beam}\left(p\right)=\sum_{n=0}^{N-1}\mathrm{exp}\left(-nd\sum_{c}\left({\overline{r} }^{c}\left(p\right)\cdot {\mu }_{E}^{c}\left(p\right)\right)\right),$$

7 $${\overline{A} }_{FL}\left(p\right)={\int }_{\Omega }d\sigma \mathrm{exp}\left(-l\left(\sigma \right)\cdot \sum_{c}\left({\overline{r} }^{c}\left(p,\sigma \right)\cdot {\mu }_{FL}^{c}\left(p, \sigma \right)\right)\right),$$

8 $${\overline{r} }^{c}\left(p\right)=\frac{1}{N}\sum_{n=0}^{N-1}{r}^{c}\left(p,n\right).$$

**Scheme 1:**
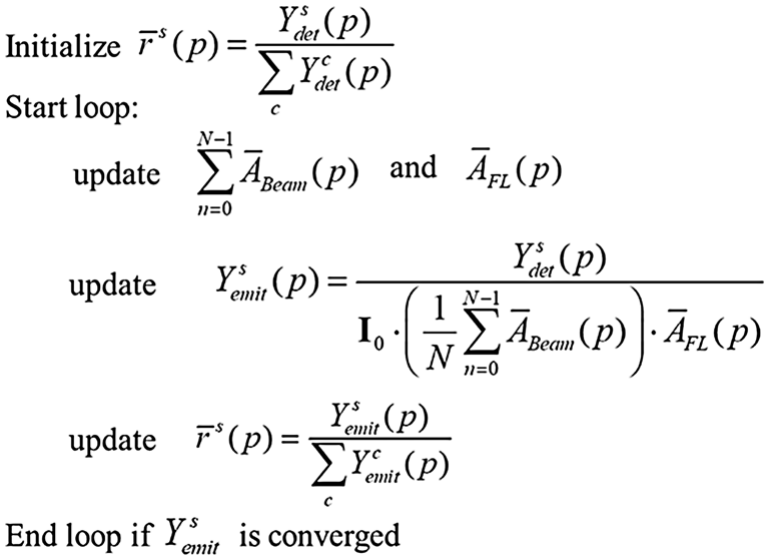
Iterative strategy for self-absorption correction.

In solving ${Y}_{emit}$, we adopt the following iterative strategy (Scheme 1) for achieving a self-consistent solution. In general, we stop the iteration when the sum of the difference of ${Y}_{emit}^{s}$ between successive iterations less than 1% for all elements *(s)* (Eq. (9)).

9 $$\frac{\mathrm{abs}\left({\sum_{p}{Y}_{emit}^{s}\left(p\right)}^{iter=k}-{\sum_{p}{Y}_{emit}^{s}\left(p\right)}^{iter=k-1}\right)}{\mathrm{abs}\left({\sum_{p}{Y}_{emit}^{s}\left(p\right)}^{iter=k}\right)}<1\%.$$

The assumption of uniform composition produces 2nd and higher-order errors to the approximation made for Eq. (5) (see “Supplementary Information”). It is also interesting to consider a situation when the material is not 100% solid, for example, with pores embedded inside. Discussion about this situation is elaborated in the Supplementary Information. We can correct the attenuation using the same protocol with a simple treatment. In the following, we will evaluate the accuracy of the recovered material composition using the iterative scheme under three categorically different cases. It is important to point out that absorption correction can be easily modified to accommodate a different measurement geometry, such as the surface normal of the sample being tilted by 45 degrees with the incidence x-ray beam.

## Results

**Figure 2:**
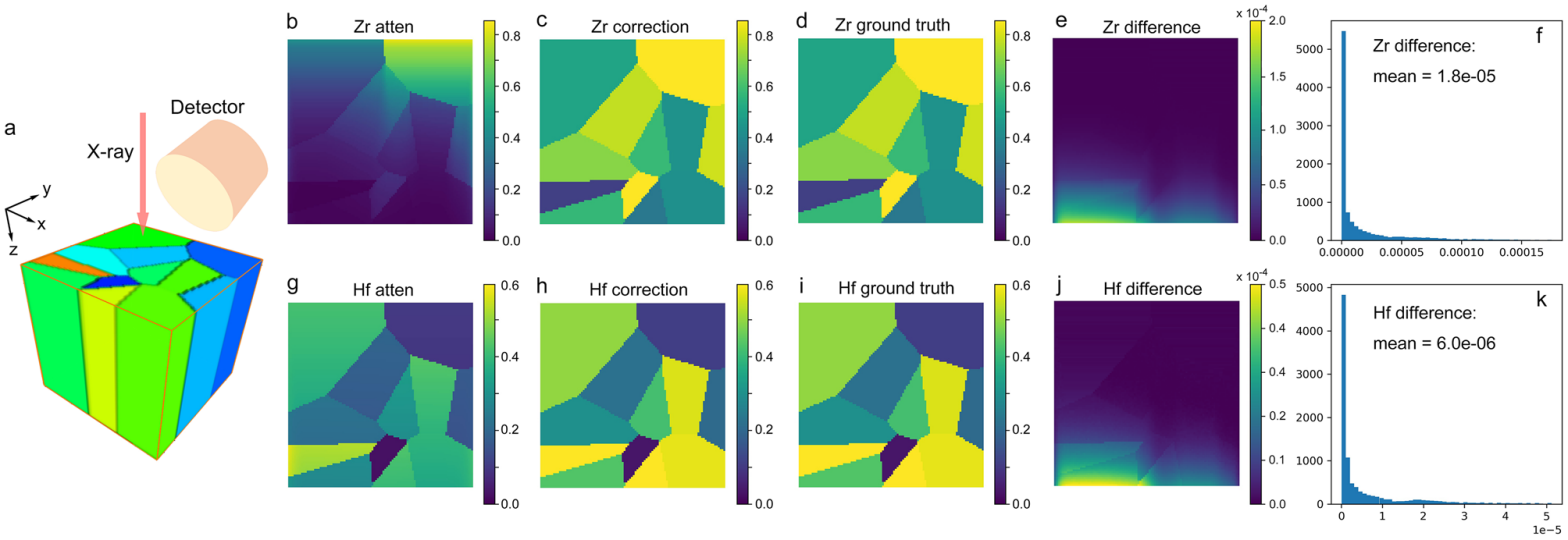
Simulation on self-absorption correction. (**a**) Zr-Hf binary cubical sample and XRF mapping setup. Composition varies in the x–y plane but is constant along the z-direction. X-ray beam illuminates the sample in the z-direction, and a raster scan is performed in the x–y plane. The cube has a size of 100 × 100 × 100 voxels with a 50 nm voxel size. (**b**) simulated 2D XRF image of Zr (**c**) XRF image of Zr after the absorption correction. (**d**) ground truth of Zr XRF image (**e**) fraction difference of Zr, calculated as: |correction-ground truth|/ground truth. (**f**) histogram of (**e**). (**g**) simulated 2D XRF image of Hf. (**h**) XRF image of Hf after the absorption correction. (**i**) ground truth of XRF of Hf XRF image. (**j**) fraction difference of Hf. (**k**) histogram of (**j**).

The first case corresponds to the system where the sample has a uniform composition along the beam direction, as illustrated in Fig. 2. We simulated a sample with a binary composition containing Zr and Hf. Table 1 summarizes the relevant parameters for the sample system, with a typical energy range for the hard X-ray fluorescence microscopy experiments, where the L_α_ emissions of Zr and Hf are detected. Note that the raster scan is conducted in the x–y plane with the incidence beam along the z-direction. The detector is positioned perpendicular to the incidence beam to minimize the scattering background and collects the XRF signal along the y-axis.

**Table 1:** Parameters used in the simulation of the Zr-Hf and Fe–Ni binary alloy systems.

	Emission energy (keV)	Mass attenuation coefficient (cm^2^/g) for elements (Zr, Hf, Fe, Ni) at energies of:		
		12 keV	2.044 keV	7.899 keV
Zr	2.044	45.09	769.44	140.35
Hf	7.899	226.87	3404.19	162.17

		12 keV	6.404 keV	7.478 keV
Fe	6.404	104.7	71.04	361.87
Ni	7.478	129.65	91.26	59.73

The *L*_*α*_ emission was used for Zr and Hf, while the *K*_*α*_ emission was used for Fe and Ni.

The simulated 2D XRF image for Zr and Hf are shown in Fig. 2b and g, respectively. The Zr image shows more significant attenuation than the Hf image due to its lower $L\alpha$ emission energy. The accuracy of the correction is evaluated by calculating the percentage error (PErr) between the intensity (${Y}_{emit}^{s}$) after the correction with respect to the ground truth value:

10 $$\mathrm{PErr}=\frac{\left|{Image}_{correction}-{Image}_{groundtruth}\right|}{{Image}_{groundtruth}}.$$

PErr is evaluated at each pixel of the Zr and Hf image, producing a PErr image for Zr and Hf, shown in Fig. 2e and j, respectively. Figure 2f and k are their image histograms showing the range of the PErr values. These simulated data show that the PErr values are very low for Zr and Hf after the correction, indicating that we can fully correct the self-absorption in the case of the uniform concentration through the sample thickness.

In case the elemental distribution varies through the sample thickness, we could consider two different possibilities. The second case corresponds to the materials with slow composition variation along the thickness. This case represents the materials with large grains whose sizes are comparable to the sample thickness (samples with thin sections) or the attenuation length of the incident X-ray beam. In the third case, the composition fluctuates significantly, corresponding to a more complex material system. In the absence of the exact knowledge of how the elements are precisely distributed, we introduce a concept of the modulation behavior of the elemental distribution using the following expression.

11 $$I\left(x,y,z\right)=\overline{I}\left(x,y\right)+\alpha \cdot cos\left(2\pi \nu z\right).$$

The first term, $\overline{I}\left(x,y\right)$, describes the constant elemental distribution. The second term describes the fluctuating elemental distribution with the modulation amplitude, $\alpha$, and the modulation frequency, $\nu$, which has a unit of 1/voxel size. In the simulation, we vary $\nu$ in a range of 0 to 20. $\nu$ $=0$ corresponds to the uniform composition. $\nu$ $=20$ represents a case where the elemental distribution varies drastically. For example, assuming a sample has a thickness of 5 um with an imaging resolution of 50 nm, $\nu$ $=20$ represents a modulation period of 100 nm, corresponding to a highly fluctuating elemental variation, which requires the state-of-the-art X-ray nanoprobe to revolve.

**Figure 3:**
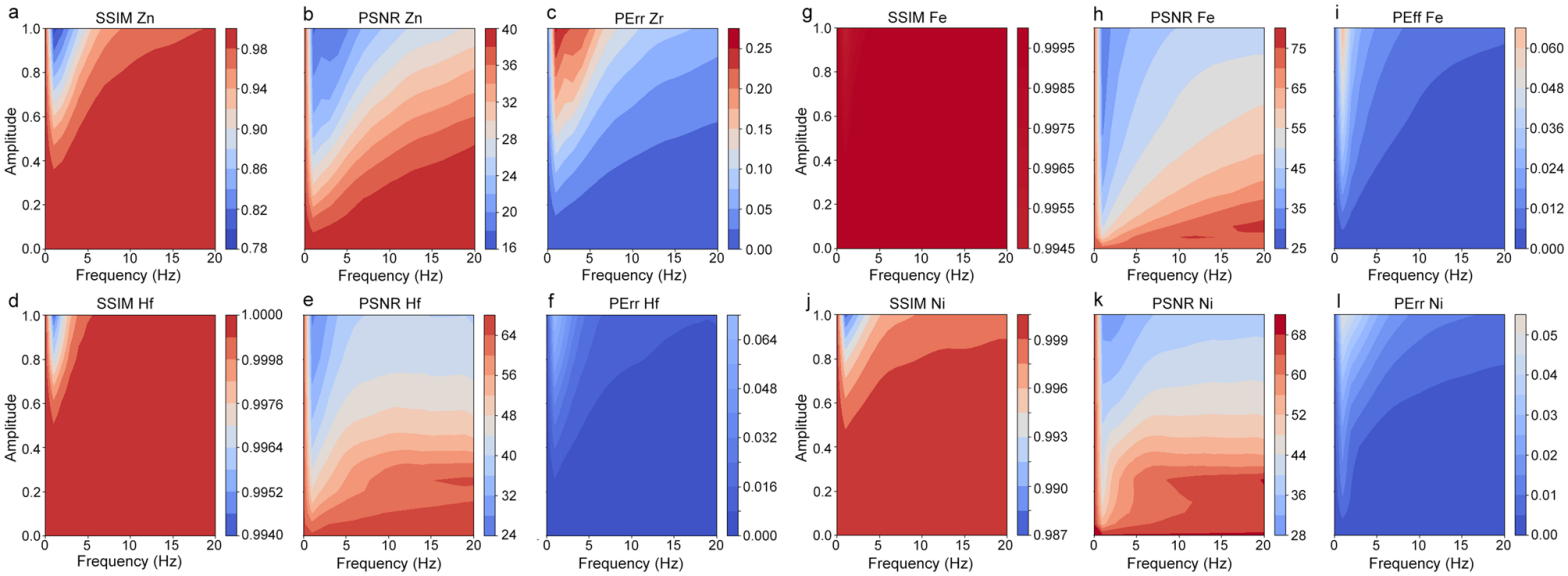
Evaluation of the absorption correction: For a Zr-Hf sample (**a-f**) and a Fe-Co sample (**g-l**) with a varying composition modulation in the z-direction. (**a**) SSIM. (**b**) PSNR. (**c**) PErr of Zr XRF image after absorption correction with respect to ground truth. (**d**) SSIM. (**e**) PSNR. (**f**) PErr of Hf XRF image after absorption correction with respect to ground truth. (**g**) SSIM, (**h**) PSNR. (**i**) PErr of Fe XRF image after absorption correction with respect to ground truth. (**j**) SSIM, (**k**) PSNR, (**l**) PErr of Ni XRF image after absorption correction with respect to ground truth.

Figure 3a–f present the correction accuracy analysis of Zr and Hf by evaluating three metrics: structural similarity index measurement (SSIM)^18^, peak signal-to-noise ratio (PSNR)^19^, and PErr. SSIM and PSNR are evaluated from the corrected and the ground truth images. PErr is evaluated over the entire XRF image taking the average value. As expected, the quality of absorption correction is poor for high modulation amplitude, indicated by small SSIM and PSNR values and high PErr values. In addition, the correction quality is excellent for the case of zero modulation frequency, which is not a surprise. An interesting result is that for a fixed modulation amplitude, for example, at 0.5, the correction quality slowly increases with the higher modulation frequency. This result can be explained in the following way. If the modulation frequency is exceptionally high, the total detector signal (often extending to many tens of degrees) is integrated over a large number of individual fluctuation spatial regions, leading to insensitive individual variation (i.e. the sample appears to be less grainy). As the modulation frequency decreases but is not equal to zero, the absorption variation over the representative spatial region increases, leading to higher correction errors (i.e. the sample appears grainier).

In comparing the correction evaluation metrics, Hf has better correction accuracy than Zr. The PErr for Hf correction is less than 0.08 (or 8%) for all simulated structures. In contrast, the PErr value for Zr (with a very low XRF emission energy) reaches above 0.25. Even for Zr, the PErr values improve as the modulation amplitude decreases for all modulation frequencies. Additional simulation with different sample geometry is reported in Fig. S1 and S2 in Supporting Information.

Hf-Zr is an example of a very challenging system due to low XRF emission through a highly absorbing matrix, which tends to give higher correction errors. In contrast, better correction results are achieved for the material with more energetic XRF emission. To illustrate this, we show a simulation from a Fe–Ni binary system. The results are shown in Fig. 3g–l. The simulation indicates that both Fe and Ni are adequately corrected with PErr values less than 0.06 for all modulation amplitudes and frequencies. A similar trend for correction improvement at a fixed modulation amplitude is also observed in this binary system. It is important to point out that our expression for the elemental modulation can be extended in a general way by Fourier decomposing the elemental variation through the thickness, T with phase shift, ${\varphi }_{n}$ as

12 $$\sum_{n=0}^{\infty }{\alpha }_{n}cos\left(\frac{2\pi nz}{T}- {\varphi }_{n}\right).$$

The aforementioned three cases are the special cases for the general Fourier decomposition. The first case corresponds to the zeroth order (n = 0), and the second and third cases correspond to a situation with a single modulation frequency with a small and large value, respectively.

We demonstrated the effectiveness of our absorption correction method by applying it to XRF mapping data collected from a corroded stainless steel (SS) sample. Corrosion in stainless steel is a major issue for many industries. The composition and uniformity of the steel are crucial for its stability in corrosive environments. Specifically, the grain boundary plays a key role in determining corrosion behavior. An in-depth study to reveal the intergranular structure on the nanoscale is important to advance our fundamental knowledge for mitigating the corrosion process. The sample we studied here is 304 SS, which was electrochemically corroded in sulfuric acid. The details are described elsewhere^20^. In 304 stainless steel (SS), Cr, Ni, and Fe are the three major components that account for ~ 97% of the total weight. For instance, Cr and Ni have a weight percentage range of 17.5–19.5% and 8–10.5%, respectively^21^. Nanoscale XRF mapping measurements were conducted at the HXN beamline at NSLS-II. The measurement details are described in the Methods section.

**Figure 4:**
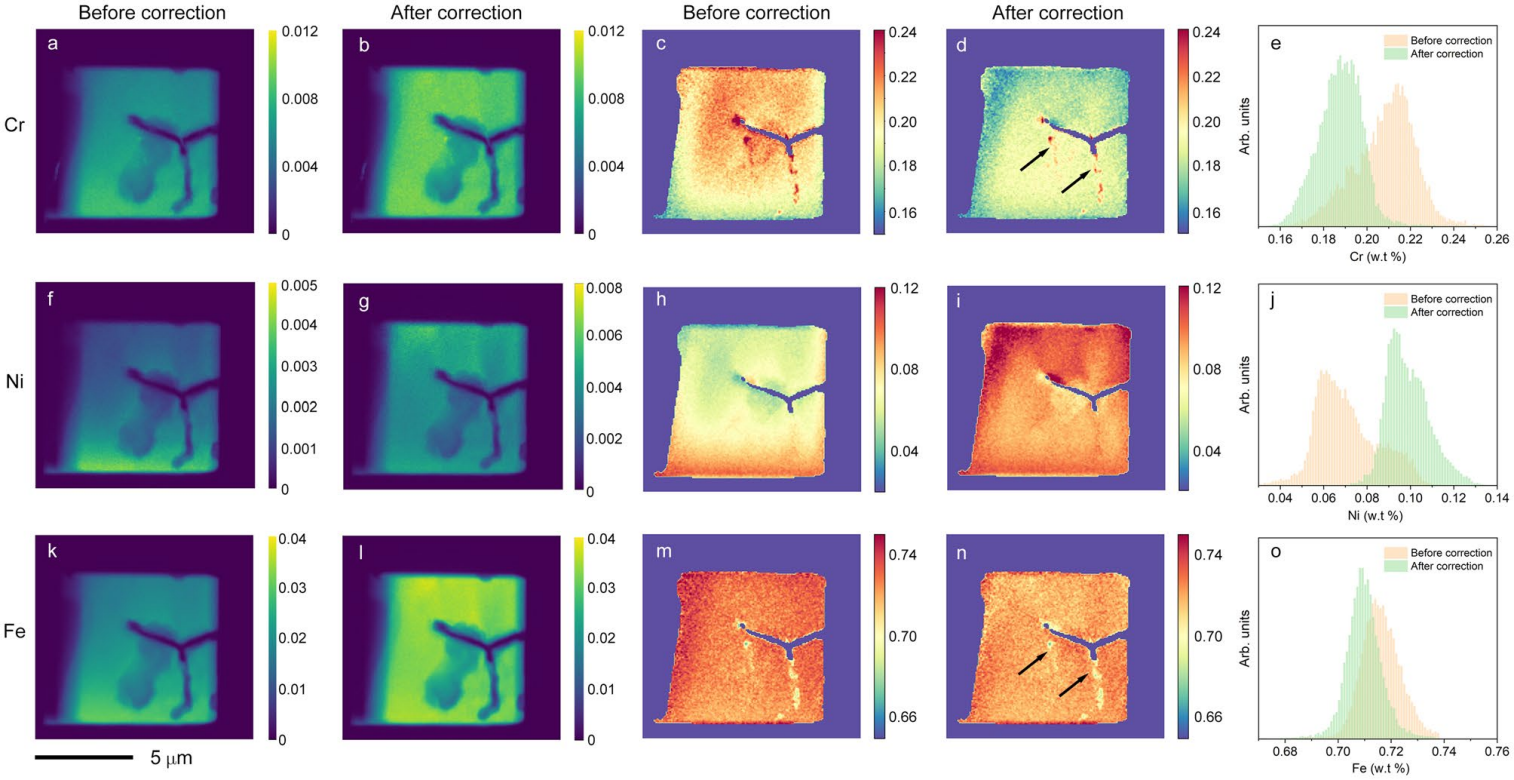
Element distributions in sensitized stainless steel. (**a**) Cr XRF image before correction. (**b**) Cr XRF image after correction, (**c**) Cr concentration (i.e. weight percentage) map before correction, (**d**) Cr concentration map after correction. Two arrows point to the localized Cr enrichment clusters along the grain boundaries (**e**) histogram of the Cr concentration map before and after correction. (**f**) Ni XRF image before correction, (**g**) Ni XRF image after correction, (**h**) Ni concentration map before correction, (**i**) Ni concentration map after correction, (**j**) histogram of the Ni concentration map before and after correction. (**k**) Fe XRF image before correction, (**l**) Fe XRF image after correction, (**m**) Fe concentration map before correction, (**n**) Fe concentration map after correction. Two arrows point to the same regions shown in n, (**o**) histogram of the Fe concentration map before and after correction. A fractional concentration of an element, for example, Cr, is obtained by Cr/(Cr + Ni + Fe) using their weight percentages. All images share the same scalebar.

Figure 4a,f and k show the “as-measured” fluorescent intensity map of Cr, Ni, and Fe. Slow variation of intensity from top to bottom is due to self-absorption. Localized low-intensity features (appearing darker) are the structural modifications (i.e. cracking) by electrochemical corrosion along the grain boundaries. While these images provide interesting nanoscale structural modification, quantitative analysis of the uncorrected data is problematic due to strong self-absorption. Consequently, the extracted fractional concentration maps in Fig. 4c,h and m are not quantitatively accurate. In the absorption-corrected images (Fig. 4b,g,l), the slow intensity variation from top to bottom is largely suppressed, revealing much finer localized features. For example, the corrected Cr concentration map exhibits highly localized Cr clusters decorated along lines indicated by arrows. The corrected concentration map for Cr, Ni, and Fe are shown in Fig. 4d,i and n. After correction, the Cr concentration map is consistent with the currently accepted corrosion mechanism. At the grain boundaries, Cr is partially phase-separated, increasing susceptibility for electrochemical corrosion for the SS matrix. After corrosion, the weakened grain boundaries are cracked and dissolved away. Some grain boundaries are not yet fully cracked, but they are decorated by lower Fe concentration and highly localized Cr clusters, as indicated by arrows in Fig. 4d and n. The histograms of the Cr, Ni, and Fe concentration maps are shown in Fig. 4e,j and o. Prior to the correction, the distribution of Cr displayed a broad range with a center value of 21.8%, which is inconsistent with the nominal range of Cr in 304 SS (17.5–19.5%). Similarly, the median composition value of Ni was not consistent with the nominal values of 8–10.5%. After correction, the concentrations of all three elements agree well with these nominal values, as expected. On the other hand, the corrective direction of the Cr concentration is counterintuitive. Since Cr suffers the most significant amount of absorption among the three elements, it is natural to think that the correction should move the median value of Cr from low to high. In addition, since Ni suffers the least amount of absorption, the correction amount for Ni should be less than that of Cr. Our data exhibit the opposite trend, even though the absorption correction recovers the correct concentration values. We attribute our findings to the secondary fluorescence effect, which is a second-order correction to the data. The secondary fluorescence for the stainless-steel sample occurs when the XRF photons from Ni produce an additional fluorescence signal from Fe and Cr, and the Fe XRF photons generate additional Cr fluorescence. Consequently, the total detected Cr signal is larger than the expected value considering the absorption only. Therefore, the absorption correction moves the median value of Cr to the correct value but in the opposite direction. Correcting the secondary fluorescence is even more computationally demanding than correcting the absorption and should be discussed in a separate publication.

## Conclusion

We propose a method for tackling the problem of self-absorption in 2D XRF mapping, which is significantly more difficult than the 3D case. The accuracy of our methodology is comprehensively evaluated at different absorption levels with different elemental combinations. We show that samples with higher fluorescence emission energies can be better recovered with errors significantly less than 10% in most cases. Application of our method to the XRF mapping experiment on the corroded stainless steel resulted in significant visibility of nanostructures and, more importantly, a more accurate description of Cr, Ni, and Fe concentration distribution. We also note that our method is applicable to the XRF mapping at the micron scale, useful for both micro-XRF and nano-XRF investigations.

## Material and method

A corroded 304 stainless steel sample was prepared by electrochemical corrosion in 1 M sulfuric acid and 0.005 potassium thiocyanate by applying a voltage of 300 mV above open-circuit potential. Details of sample preparation can be found elsewhere^19^.

Nanoscale XRF mapping measurements were performed at the Hard X-ray Nanoprobe (HXN) Beamline at 3-ID of the NSLS-II^22^. Monochromatic X-rays with an energy of 12 keV were focused by a Fresnel zone plate with a 40 nm outermost zone. XRF mapping was acquired in a fly-scan mode with a dwell time of 0.05 s/pixel. The pixel resolution of 80 nm was used for XRF mapping. The XRF spectra were analyzed by PyXRF^23^ to produce raw elemental images. Then, the absorption correction was performed using algorithms written in Python.

## Supplementary Information

Supplementary Information.

## Data Availability

All data needed to evaluate the conclusions in the paper are present in the paper or the Supplementary Materials. Additional data related to this paper may be requested from the authors.
