# Supplementary material for: Self-absorption correction on 2D X-ray fluorescence maps
Source: Sci Rep. 2023 May 4;13:7271. doi: 10.1038/s41598-023-33383-w (PMC10160062; doi:10.1038/s41598-023-33383-w)
Supplement: Supplementary file 1 — Supplementary Information. [file 41598_2023_33383_MOESM1_ESM.docx]

# Self-absorption correction on 2D X-ray fluorescence maps

Mingyuan Ge, Hanfei Yang, Xiaojing Huang, Yong S. Chu

National Synchrotron Light Source II, Brookhaven National Laboratory, Upton, NY, USA

**I. Derivation of Eq.5 in the main text**

All the notations are defined in the main text.

(S1) $Y_{det}^{s}(p)=I_{0}\sum_{n=0}^{N-1} \left[A_{Beam}(p,n)\cdot Y^{s}(p,n)\cdot A_{FL}(p,n)\right]$

Assuming the material composition is homogeneous in the z-direction $r^{c}(p,n)\to\bar{r}^{c}(p)$, and substituting$A_{Beam}(p,n)$with Eq. 2 in the main text, then we get:

(S2) $Y_{det}^{s}(p)\approx I_{0}\sum_{n=0}^{N-1} \left[A_{Beam}(p,n)\cdot\bar{Y}^{s}(p)\cdot\bar{A}_{FL}(p)\right]$

$$=I_{0}\cdot\bar{Y}^{s}(p)\cdot\bar{A}_{FL}(p)\cdot\sum_{n=0}^{N-1} \exp\left(-nd\sum_{c} \left(\bar{r}^{c}(p)\cdot\mu_{E}^{c}(p)\right)\right)$$

$$=I_{0}\cdot\bar{Y}^{s}(p)\cdot\bar{A}_{FL}(p)\cdot\sum_{n=0}^{N-1} \bar{A}_{Beam}(p)$$

Since:

(S3) $\bar{Y}^{s}(p)=\frac{1}{N}\sum_{n=0}^{N-1} Y^{s}(p,n)$

$Y_{det}^{s}(p)$is accurately expressed by:

(S4) $Y_{det}^{s}(p)\approx I_{0}\cdot\left(\frac{1}{N}\sum_{n=0}^{N-1} \bar{A}_{Beam}\right)\cdot Y_{signal}(p,n)$

with

(S5) $Y_{signal}(p,n)=\left(\sum_{n=0}^{N} \left[Y^{s}(p,n)\cdot\int_{\Omega} d\sigma\exp\left(-l(\sigma)\cdot\sum_{c} \left(r^{c}(p,\sigma)\cdot\mu_{FL}^{c}(p,\sigma)\right)\right)\right]\right)$

For simplicity, we drop all the coordinate notation of *p*:

(S6) $Y_{signal}=\left(\sum_{n=0}^{N} \left[Y^{s}\cdot\int_{\Omega} d\sigma\exp\left(-l\cdot\sum_{c} \left(r^{c}\cdot\mu_{FL}^{c}\right)\right)\right]\right)$

Switching the integration and summation order, we get the following:

(S7) $Y_{signal}=\int_{\Omega} d\sigma\left(\sum_{n=0}^{N} \left[Y^{s}\cdot\exp\left(-l\cdot\sum_{c} \left(r^{c}\cdot\mu_{FL}^{c}\right)\right)\right]\right)$

Taylor expansion of the exponential function gives eq.(S8), where R is the high-order terms of the expansion:

(S8) $Y_{signal}^{s}=\int_{\Omega} d\sigma\left(\sum_{n=0}^{N-1} Y^{s}\left(1-l\cdot\sum_{c} (r^{c}\cdot\mu_{FL}^{c})+R\right)\right)$

Expanding the equation:

(S9) $Y_{signal}^{s}=\int_{\Omega} d\sigma\left(\sum_{n=0}^{N-1} Y^{s}-\left(l\cdot\sum_{c} \mu_{FL}^{c}\sum_{n=0}^{N-1} \left(Y^{s}\cdot r^{c}\right)+\sum_{n=0}^{N-1} Y^{s}\cdot R\right)\right)$

By defining:

(S10) $Y^{s}=\bar{Y}^{s}+\Delta_{n}\text{, }r^{c}=\bar{r}^{c}+\delta_{n}$

We write:

(S11) $Y_{signal}^{s}=\int_{\Omega} d\sigma\left(\sum_{n=0}^{N-1} Y^{s}-\left(l\cdot\sum_{c} \mu_{FL}^{c}\sum_{n=0}^{N-1} \left[\left(\bar{Y}^{s}+\Delta_{n}\right)\cdot\left(\bar{r}^{c}+\delta_{n}\right)\right]+\sum_{n=0}^{N-1} Y^{s}\cdot\text{R}\right)\right)$

(S12) $∵\sum_{n=0}^{N-1} \Delta_{i}=0\text{ and }\sum_{n=0}^{N-1} \delta_{n}=0$

$$\therefore\sum_{n=0}^{N-1} \left[(\bar{Y}^{s}-\Delta_{n})\cdot(\bar{r}^{c}-\delta_{n})\right]=\sum_{n=0}^{N-1} \left(\bar{Y}^{s}\cdot\bar{r}^{c}\right)+\sum_{n=0}^{N-1} \left(\Delta_{n}\cdot\delta_{n}\right)$$

By using:

(S13) $\sum_{n=0}^{N-1} Y^{s}=N\cdot\bar{Y}^{s}$

and denoting:

(S14) $\alpha=\sum_{n=0}^{N-1} \left(\Delta_{n}\cdot\delta_{n}\right)$

we rewrite the equation to:

(S15) $Y_{signal}^{s}=\int_{\Omega} d\sigma\left[N\cdot\bar{Y}^{s}-N\cdot\bar{Y}^{s}\cdot l\cdot\sum_{c} \left(\mu_{FL}^{c}\cdot\bar{r}^{c}\right)-l\cdot\sum_{c} \left(\mu_{FL}^{c}\cdot\alpha\right)+N\cdot\bar{Y}^{s}\cdot R\right]$

Assumptions:

1. The detector has a small acceptance angle, e.g., $\theta_{\max}=40^{o}$ vertically and horizontally (refer to HXN beamline at NSLS-II). The maximum value of $l_{\max}$is:

Therefore, we can approximate $l$as a constant for all angles within the solid angle defined by the detector camera.


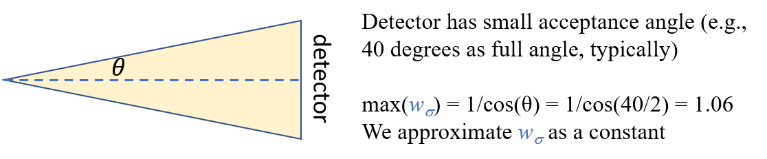


1. For each location in the 2D scan plane, we assume a relatively small compositional variation in the z-direction, which gives $\alpha=\sum_{i}^{n} \left(\Delta_{i}\cdot\delta_{i,s}\right)\approx0$

It turns out:

(S16) $Y_{signal}^{s}=\int_{\Omega} d\sigma\left[N\cdot\bar{Y}^{s}-N\cdot\bar{Y}^{s}\cdot l\cdot\sum_{c} \left(\mu_{FL}^{c}\cdot\bar{r}^{c}\right)+N\cdot\bar{Y}^{s}\cdot R\right]$

(S17) $Y_{signal}^{s}\approx\left(N\cdot\bar{Y}^{s}\right)\cdot\int_{\Omega} d\sigma\left(1-l\cdot\sum_{c} \left(\mu_{FL}^{c}\cdot\bar{r}^{c}\right)+R\right)$

Reverse the Taylor expansion of the exponential function:

(S18) $Y_{signal}^{s}\approx\left(N\cdot\bar{Y}^{s}\right)\exp\left(\int_{\Omega} d\sigma\left(-l\cdot\sum_{c} \left(\mu_{FL}^{c}\cdot\bar{r}^{c}\right)\right)\right)$

$$=Y_{emit}^{s}\cdot\exp\left(\int_{\Omega} d\sigma\left(-l\cdot\sum_{c} \left(\mu_{FL}^{c}\cdot\bar{r}^{c}\right)\right)\right)$$

Substitute (S18) into (S4) gives equation (S19), which is the same equation as **Eq.5** in the main text.

(S19) $Y_{det}^{s}(p)\approx I_{0}\cdot\left(\frac{1}{N}\sum_{n=0}^{N-1} \bar{A}_{Beam}\right)\cdot Y_{emit}^{s}(p)\cdot\exp\left(\int_{\Omega} d\sigma\left(-l\cdot\sum_{c} \left(\mu_{FL}^{c}\cdot\bar{r}^{c}\right)\right)\right)$

$$=I_{0}\cdot\left(\frac{1}{N}\sum_{n=0}^{N-1} \bar{A}_{Beam}\right)\cdot Y_{emit}^{s}(p)\cdot\bar{A}_{FL}(p)$$

**II.** **Additional simulation results**

At a location where the sample has reduced local thickness or internal voids, a lower fluorescence signal is produced, which can be normalized by a factor,

(S20) $\alpha=\frac{\sum_{s} Y_{det}^{s}\left(p\right)}{\max\left(\sum_{s} Y_{det}^{s}\left(p\right)\right)}$

Correspondingly, the number of pixels at position (p) is normalized to:

(S21) $N\left(p\right)=\alpha N_{\max}$

(S19) can be updated to:

(S22) $Y_{det}^{s}\left(p\right)=I_{0}\cdot\left(\frac{1}{N\left(p\right)}\sum_{n=0}^{N\left(p\right)-1} \bar{A}_{Beam}\right)\cdot Y_{emit}^{s}(p)\cdot\bar{A}_{FL}(p)$

In this case, there is freedom in constructing the thickness profile of $N\left( p \right)$ in 3D. One choice is assuming a central symmetric distribution of $N\left( p \right)$. For example, **Fig. S1** demonstrates a simulation of the absorption correction on a binary Zr-Hf alloy sample with a spherical shape geometry. The simulated volume is 100 x 100 x 100 voxels with a voxel size of 50 nm. The energy of the incidence X-ray is 12 keV. The Zr and Hf composition is randomly initialized between (0, 1) for each grain. We simulated a total of 40 different compositions to evaluate the overall performance of the proposed self-absorption algorithm. **Fig. S1** shows one of the simulated compositions. The average of the correction error is 0.05 for Zr and 0.01 for Hf.

Notice that the thickness profile can be more accurately approximated if additional geometry information is available. For example, from multiple tilted views using scanning electron microscopy (SEM) or a tomographic reconstruction from transmission X-ray microscopy (TXM), which can be performed in a very short time (e.g., 1 minute) in the state-of-the-art TXM synchrotron beamline [1, 2].


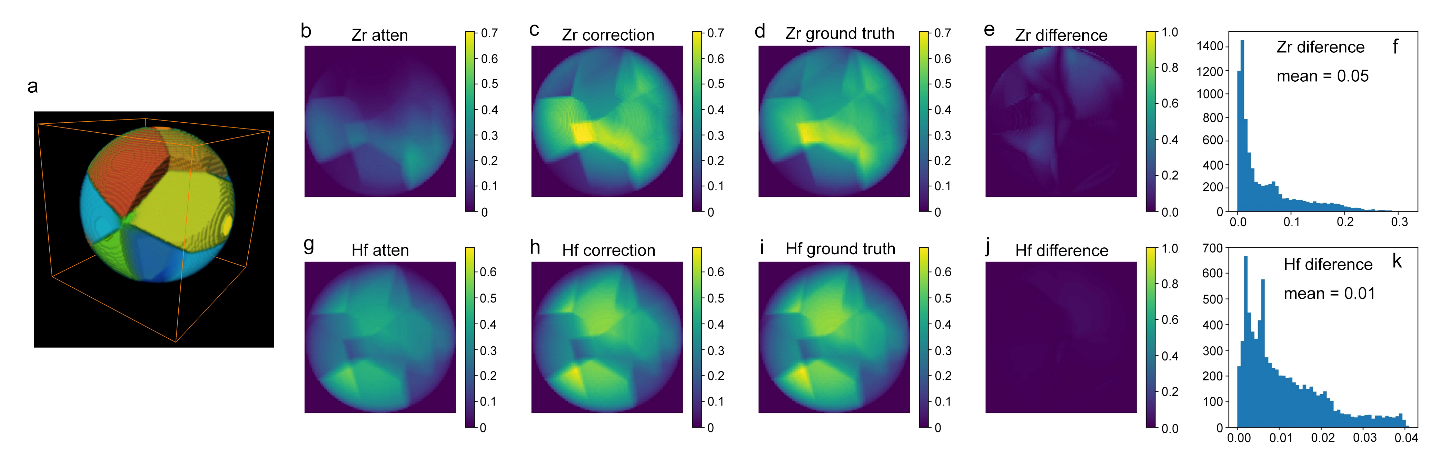


**Figure S1.** **Simulation on self-absorption correction.** **(a)** Zr-Hf binary spherical sample and XRF imaging setup. **(b)** simulated 2D XRF image of Zr **(c)** XRF image of Zr after the absorption correction. **(d)** ground truth of Zr XRF image **(e)** fraction difference of Zr, calculated as: |correction-ground truth| /ground truth. **(f)** histogram of **(e)**. **(g)** simulated 2D XRF image of Hf. **(h)** XRF image of Hf after the absorption correction. **(i)** ground truth of XRF of Hf XRF image. **(j)** fraction difference of Hf. **(k)** histogram of **(j)**.

**Fig. S2** summarizes the PSNR and SSIM for all 40 compositions. After correction, the PSNR increases from ~10 to ~28 for Zr and from ~20 to ~45 for Hf. The SSIM increases from ~0.4 to ~0.95 for Zr and from ~0.95 to ~0.99 for Hf.


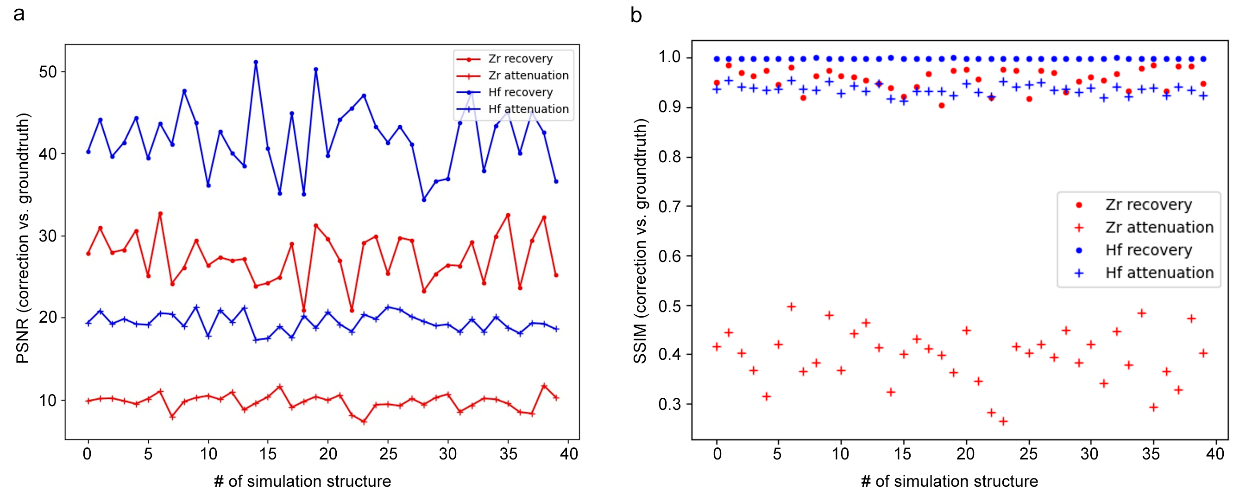


**Figure S2.** Evaluation of PNSR **(a)** and SSIM **(b)** of the simulated structure before and after absorption correction with respect to ground truth.

[1] Mingyuan Ge, David Scott Coburn, Evgeny Nazaretski, Weihe Xu, Kazimierz Gofron, Huijuan Xu, Zhijian Yin, and Wah-Keat Lee, “One-minute nano-tomography using hard X-ray full-field transmission microscope”, *Appl. Phys. Lett*. **2018**, *113*, 083109

[2] Jiayong Zhang, Wah-Keat Lee, Mingyuan Ge, “Sub-10 second fly-scan nano-tomography using machine learning”, *Communications Materials,* **2022**, *3*, 91
